# Reevaluating the Causes of Otitis Externa: A Case Report

**DOI:** 10.7759/cureus.74340

**Published:** 2024-11-24

**Authors:** Kelvin Yong Jie Lim, Soon Heng Tan

**Affiliations:** 1 Department of Otorhinolaryngology, Khoo Teck Puat Hospital, Singapore, SGP; 2 Department of Otorhinolaryngology, Woodlands Health Campus, Singapore, SGP

**Keywords:** acute otitis externa, external ear, infectious and parasitic diseases, mites, otoacariasis

## Abstract

A 28-year-old female domestic helper presented to the Ear, Nose, and Throat clinic complaining of three weeks of right otalgia associated with a right blocked ear. The hearing was otherwise normal, and she denied otorrhoea, dizziness or imbalance, ear digging, or water contact, and has no history of ear eczema. She has no other past medical history and no recent travel history. On examination, numerous whitish ovoid lesions were seen lining the entire right external auditory canal (EAC), admixed with debris, and the canal was inflamed. Her tympanic membrane was intact. The contralateral ear was normal. She was diagnosed with otoacariasis. The ear mites were unable to be retrieved for a direct microscopic examination, but based on the ovoid, smooth, and translucent morphology of the mites, they likely belong to the Acaridae or Chortoglyphidae families of mites. She underwent aural toileting for complete removal of the ear mites and was prescribed ear drops containing dexamethasone and polymyxin B sulfate for 2 weeks. She was reviewed using the otomicroscopy technique two weeks later and had made an uneventful recovery with no recurrence of ear mites.

## Introduction

Otoacariasis, the infestation of the ear canal by ticks or mites, is a rare but important condition encountered in human and veterinary medicine. While it is more commonly reported in animals, especially in rural or agricultural settings, human cases, although infrequent, can present significant clinical challenges. The condition is characterized by the presence of live or dead arthropods within the external auditory canal (EAC), often causing inflammation and a range of otological symptoms. Early diagnosis and management are critical to prevent complications such as secondary infections, tympanic membrane (TM) damage, or persistent symptoms.

The global distribution of mites, including species from the Acaridae and Chortoglyphidae families, means that otoacariasis can occur in diverse environments, from rural areas to urban households. Transmission typically occurs through direct contact with animals, exposure to contaminated environments, or occasionally waterborne sources. Clinicians need to maintain a high index of suspicion, especially in patients presenting with unilateral otalgia, pruritus, and evidence of foreign bodies in the ear.

This case report discusses the clinical presentation, diagnosis, and management of a 28-year-old female domestic helper who presented with otoacariasis caused by suspected Acaridae mites. It highlights the importance of detailed otological examination, appropriate treatment approaches, and the role of Ear, Nose, and Throat (ENT) specialists in ensuring successful outcomes. The discussion also reviews the differential diagnoses, treatment options, and the broader implications of this uncommon condition for clinical practice and public health.

## Case presentation

**Figure 1:**
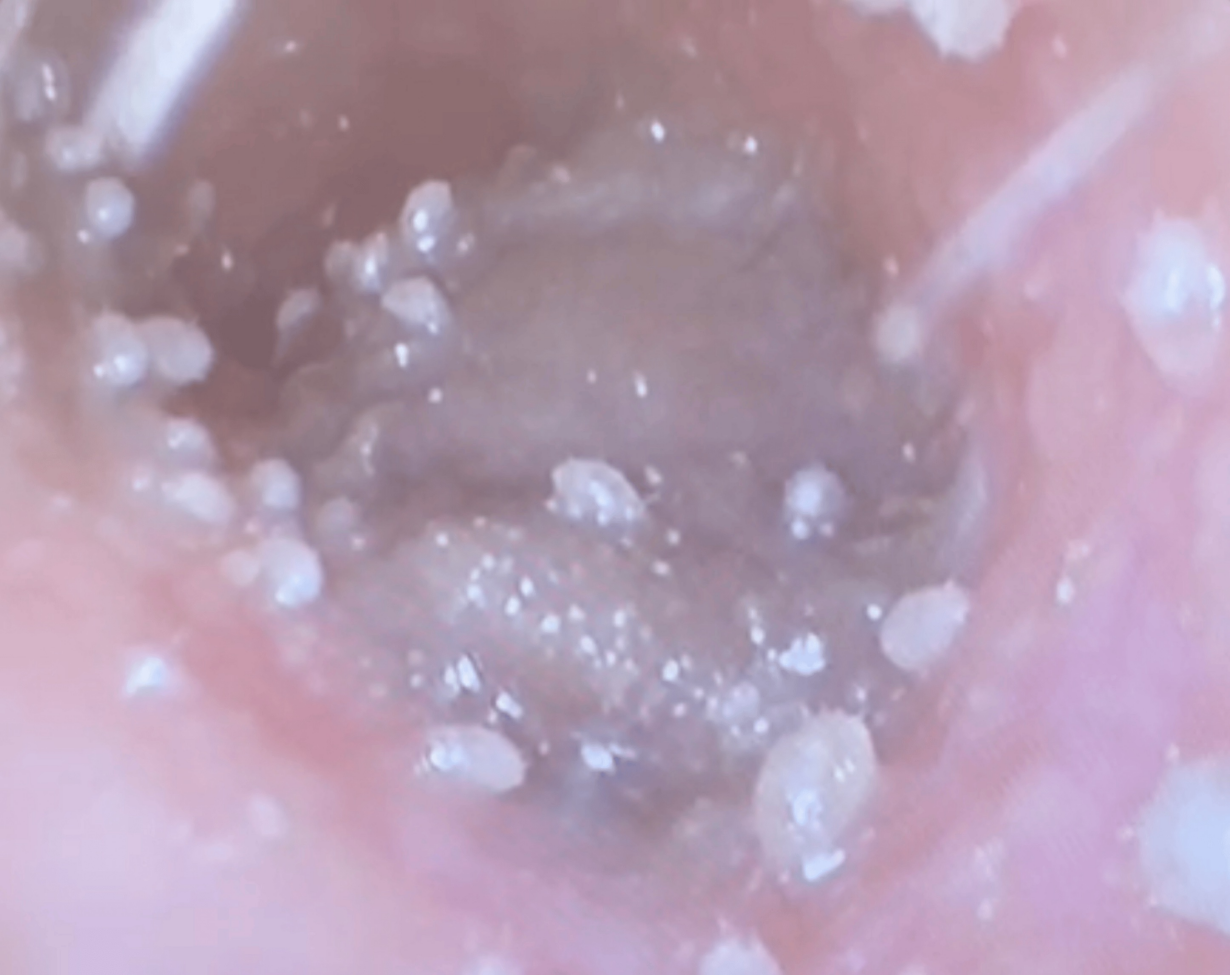
Endoscopic photograph depicting numerous whitish ovoid lesions with debris lining the right external auditory canal

A 28-year-old female domestic helper presented to the ENT clinic complaining of three weeks of right otalgia associated with a right blocked ear. The hearing was otherwise normal, and she denied otorrhoea, dizziness or imbalance, ear digging, or water contact, and has no history of ear eczema. She has no other past medical history and no recent travel history. On examination, numerous whitish ovoid lesions were seen lining the entire right EAC, as seen in Figure 1, admixed with debris, and the canal was inflamed. Video 1 depicts live mites. Her TM was intact. The contralateral ear was normal. A thorough physical examination was also conducted, revealing no evidence of mites in other areas.

**Video 1:** Endoscopic video of mites seen moving in the external auditory canal

She was diagnosed with otoacariasis. The ear mites were unable to be retrieved for a direct microscopic examination, but based on the ovoid, smooth, and translucent morphology of the mites, they likely belong to the Acaridae or Chortoglyphidae families of mites. She underwent aural toileting for complete removal of the ear mites and was prescribed ear drops containing dexamethasone and polymyxin B sulfate for two weeks to address the issue of a possible superimposed bacterial infection. She was reviewed using the otomicroscopy technique two weeks later and had made an uneventful recovery with no recurrence of ear mites.

## Discussion

Otoacariasis is a condition characterized by the presence or attachment of ticks and mites within the ear canal of humans and animals. Antonelli et al. opined that this condition is more prevalent in rural areas [1] and is a leading cause of otitis externa in dogs and cats. Van der Gaag found that otoacariasis is often associated with ceruminous gland hyperplasia [2], which results in intense itching, causing affected animals to scratch their ears and shake their heads vigorously. Farkas et al. found that the most common transmission mode is through direct contact with other animals, especially those within the same group or colony [3]. Other possible routes of infestation include exposure to mites while swimming in natural water sources, such as lakes or ponds [4].

In the case of our patient, the morphology of the mites suggests an infestation by Acaridae, a storage mite species frequently found in house dust around the world, potentially leading to exposure in domestic environments, as described in the study by O’hehir et al. [5].

Clinically, initial symptoms of otoacariasis typically include ear itching that progresses to increasing pain without any discharge (otorrhea). These symptoms should prompt a thorough examination of the ear for foreign bodies. Differential diagnoses to consider include otomycosis, which usually presents with whitish or black spores or the characteristic appearance of fungal mats and fruiting bodies under microscopy [6], and tuberculous otitis externa, although this is an extremely rare condition that is infrequently documented in the medical literature [7].

Currently, there is no universally established treatment for otoacariasis. Various treatment approaches have been tried, ranging from scabicidal agents to 2% acetic acid otic solutions [8]. Insects can cause severe local inflammatory reactions, which may account for the symptoms observed in otoacariasis. If an otoscopic examination raises suspicion of an insect, immediate irrigation of the ear canal should be performed using antibiotic eardrops, especially if there are signs of concurrent infection or inflammation. It is crucial to avoid using caustic or ototoxic eardrops if there is any suspicion of a perforated TM to prevent sensorineural hearing loss. Irrigation is beneficial not only for killing insects but also for potentially dislodging them to facilitate their removal.

Referral to an ENT specialist is essential for definitive management, as microscopic removal of the mites or ticks is often required. This is particularly important if the insect is located on the TM, where increased care is necessary to minimize the risk of accidental perforation. After removal, the presence of intact or ruptured hemorrhagic blebs is not uncommon. It is important to conduct serial examinations following removal to monitor for secondary otitis externa or the appearance of tick/mite larvae from unhatched eggs [9].

## Conclusions

In conclusion, otoacariasis is a rare but significant clinical finding. Although uncommon, it should be readily identified by ENT surgeons through careful examination and a high index of suspicion. Prompt diagnosis and management are crucial to prevent complications and ensure optimal patient outcomes. As such, ENT professionals should be well-acquainted with the presentation and treatment of this condition.

## References

[1] Antonelli PJ, Ahmadi A, Prevatt A (2001). Insecticidal activity of common reagents for insect foreign bodies of the ear. Laryngoscope.

[2] van der Gaag I (1986). The pathology of the external ear canal in dogs and cats. Vet Q.

[3] Farkas R, Germann T, Szeidemann Z (2007). Assessment of the ear mite (Otodectes cynotis) infestation and the efficacy of an imidacloprid plus moxidectin combination in the treatment of otoacariosis in a Hungarian cat shelter. Parasitol Res.

[4] Al-Arfaj AM, Mullen GR, Rashad R, Abdel-Hameed A, OConnor BM, Alkhalife IS, Dute RR (2007). A human case of otoacariasis involving a histiostomatid mite (Acari: Histiostomatidae). Am J Trop Med Hyg.

[5] O’hehir RE, Holgate ST, Aziz Sheikh (2017). Middleton’s Allergy Essentials. https://www.sciencedirect.com/book/9780323375795/middletons-allergy-essentials.

[6] Ho T, Vrabec JT, Yoo D, Coker NJ (2006). Otomycosis: clinical features and treatment implications. Otolaryngol Head Neck Surg.

[7] DeSimone DC, Heaton PR, Neff BA, Dao LN, Wengenack NL, Fadel HJ (2017). A rare case of chronic otitis externa due to Mycobacterium tuberculosis. J Clin Tuberc Other Mycobact Dis.

[8] Abi-Akl P, Haddad G, Zaytoun G (2017). Otoacariasis: an infestation of mites in the ear. Ann Clin Case Rep.

[9] Fong PY, Yong JS, Koh LH (2022). An unusual case of ear pain in a child. Ann Acad Med Singap.

